# Impacts of oxidative stress on bovine sperm function and subsequent *in vitro* embryo development

**DOI:** 10.21451/1984-3143-AR2018-0041

**Published:** 2018-08-03

**Authors:** Heinrich Bollwein, Lilli Bittner

**Affiliations:** 1 Clinic of Reproductive Medicine, Department for Farm Animals, Vetsuisse Faculty, University of Zurich, Zurich, Switzerland; 2 Clinic for Ruminants and Swine, Faculty of Veterinary Medicine, University of Leipzig, Leipzig, Germany

**Keywords:** embryo development, Oxidative stress, sperm function.

## Abstract

Low levels of reactive oxygen species (ROS) in sperm are essential for various sperm functions such as capacitation, hyperactivation and acrosome reaction. However, increased synthesis of ROS or a disruption of antioxidative status (e.g. in cryopreserved sperm) can induce oxidative stress (OS). Sperm are particularly vulnerable to OS, as their plasma membrane contains large amounts of polyunsaturated fatty acids and they have limited antioxidative capacity (due to low cytoplasmic volume). Oxidative stress disturbs sperm function by damaging sperm proteins, lipids and DNA. Under relatively low OS sperm may retain their fertilizing ability, which might result in transfer of impaired paternal molecules (e.g. damaged DNA) to the fertilized oozyte. Oocytes can repair damaged paternal DNA, but only to a certain extent. Most embryos are either repaired (based on limited DNA damage in blastocysts) or eliminated (based on low percentage of blastocyst formation when sperm with damaged DNA is used for fertilization). However, some blastocysts had increases in both DNA damage and apoptosis, which could have important implications for subsequent development. In several studies, exogenous antioxidants improved quality of sperm exposed to oxidative stress and subsequent embryo development. However, there is still a knowledge gap regarding whether these alterations affect embryonic survival and further development to a live fetus and healthy offspring.

## Introduction

Oxidative stress (OS) has a major role in pathophysiology of nearly all biological systems. MacLeod (1943) was apparently the first to report toxic effects of O_2_ on sperm. He noted in experiments carried out *in vitro* that increased O_2_ concentrations in seminal fluid hastened reductions in sperm motility and suggested that H_2_O_2_, generated by cells from O_2_, was the actual toxic agent. Three years later, Tosic and Walton (1946) described deleterious effects of H_2_O_2_ on bovine sperm motility and viability. Currently, there is no doubt that OS decreases sperm quality and male fertility *in vivo* (Agarwal and Saleh, 2002; Aitken and Baker, 2006).

## Nature of reactive oxygen species and oxidative stress

Reactive oxygen species (ROS) is a collective term that includes oxygen radicals (e.g. superoxide radical and hydroxyl radical) as well as highly reactive derivates of O_2_ that do not contain unpaired electrons (non-radicals), including hydrogen peroxide (H_2_O_2_), singlet oxygen (O_2_) and hypochlorous acid (HOCl) (Halliwell and Gutteridge, 1989).

ROS can be generated both outside and inside cells. Intracellularly, ROS can be produced by enzymes and non-enzymatically. NADPH oxidases in the cell membrane and cytochrome P450-dependent oxygenases in mitochondria and endoplasmatic reticulum are the main enzymatic sources. In addition to membrane- associated oxidases, soluble enzymes, including amino oxidases, xanthine oxidase, aldehyde oxidase, flavoprotein dehydrogenase and tryptophan dioxygenase, can also generate ROS (Freeman and Capro, 1982).

Non-enzymatic production of ROS occurs when an electron is directly transferred to oxygen. This occurs, for example, in the mitochondrial electron transport chain, where redox centers leak electrons that are accepted by oxygen to form superoxide, which is then quickly dismutated to H_2_O_2_ (Loschen *et al*., 1974; Liu *et al*., 2002). It is now widely accepted that mitochondria are the main source of intracellular ROS. A sophisticated antioxidative system counteracts and regulates ROS homeostasis. Antioxidative enzymes, including superoxide dismutase (SOD), catalase, and glutathione peroxidase (GPx), are primary compounds providing antioxidative defense. In addition, a number of low-molecular-weight antioxidants such as zinc, ascorbate, tocopherol, pyruvate, flavonoids, carotenoids, and glutathione also contribute to total antioxidative capacity (Finkel *et al*., 2000). Oxidative stress denotes a condition where ROS production overwhelms antioxidative capacity (Sies, 1993).

## Physiological roles of reactive oxygen in semen

After ejaculation, sperm undergo a series of physiological changes such as capacitation and acrosome reaction in the female genital tract (Austin and Bishop 1958; Hunter and Rodriguez Martinez, 2004). *In vitro* studies demonstrated that exogenous O ^·-^ and H_2_O_2_ promote capacitation and the acrosome reaction, whereas appropriate antioxidants prevent them (De Lamirande *et al*., 1997). Furthermore, low levels of NO^·^ promote capacitation of human sperm (Zini *et al*., 1995) as well as hyperactivation and zona pellucida binding of sperm (De Lamirande *et al*., 1997; Sengoku *et al*., 1998). Therefore, it has been demonstrated that capacitation and the acrosome reaction are redox- regulated (free radical regulated) processes enabling sperm to fertilize an ovum (reviewed by Aitken, 2017).

## Oxidation sources in semen

**Figure 1:**
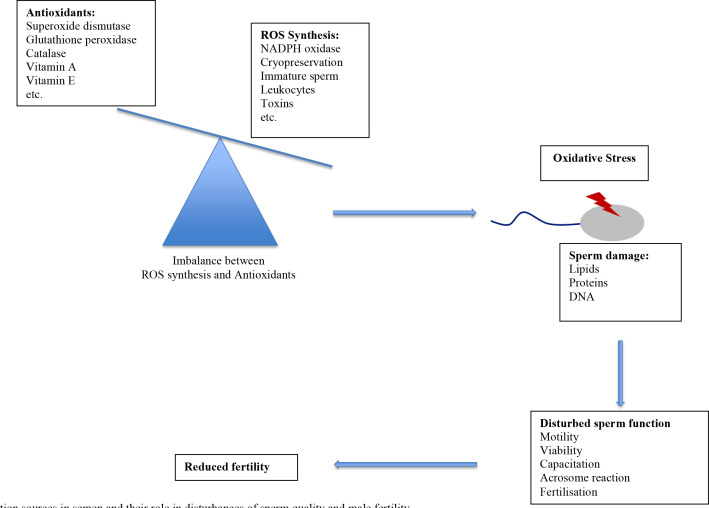
Oxidation sources in semen and their role in disturbances of sperm quality and male fertility.

The major reason for incidence of OS in semen (Fig. 1) is depletion of seminal antioxidants and excess generation of free radicals by sperm (Wathes *et al*., 2007). In cattle semen cryopreservation is well known to cause excessive production of ROS (Bilodeau *et al*., 2000) and to decrease antioxidative activity (Gürler *et al*., 2016). Free radicals appear to have important roles in cell damage after freezing and thawing, as antioxidant supplementation improved the quality of cryopreserved sperm (reviewed by Agarwal and Majzoub, 2017). However, mechanisms that increase oxidative stress in frozen-thawed sperm are not yet clear. While some authors attributed them to depletion of antioxidative enzymes (Bilodeau *et al*., 2000; Stradaioli *et al*., 2007) others propose that osmotic stress during sperm freezing and thawing induces oxidative stress (McCarthy *et al*., 2010). In somatic cells, hyperosmotic- induced cell swelling may activate membrane- associated phospholipase A2, which causes formation of free polyunsaturated fatty acids as arachnoic acid, which subsequently activates NADPH oxidase, thereby increasing O_2_ production (Lambert *et al*., 2006).

Immature sperm may produce substantial amounts of ROS that are negatively correlated with sperm quality (Ollero *et al*., 2001; Agarwal and Saleh, 2002).

There are also age-related differences in oxidative stress in bulls. For example, compared to older Simmental bulls, young bulls of the same breed were more sensitive to a decrease in sperm motility during summer, when the enzymatic antioxidative protection in seminal plasma and spermatozoa were insufficient to counteract the intensive oxidative processes in spermatozoa (Vince *et al*., 2018). Furthermore, there an effect of breed concerning the effect of heat stress on sperm quality under tropical conditions has been described. Nichi et al. (2006) noticed a higher oxidative sperm damage in Simmental and Nelore bulls during summer compared to winter, but lipid peroxidation was higher in Simmental bulls than in Nelore bulls. The authors mentioned that the GPx activity in semen of Simmental bulls might have been insufficient to avoid sperm damage that occurred concurrent with increased ROS production in summer. Our own group (Gürler *et al*. 2015) has shown that there are also inter-individual differences in OS within the same breed. We examined frozen thawed semen in nine Holstein-Friesian bulls and detected differences of the total antioxidant capacity (TAC) in seminal plasma between the bulls. The TAC values were negatively related to lipid peroxidation of sperm.

Peroxidase-positive leukocytes (mainly polymorphonuclear leukocytes and macrophages) are other sources of ROS in semen (Ochsendorf and Fuchs, 1997). Additionaly, various exogenous factors are described, which either directly induce oxidative stress in sperm or stimulate endogenous production of ROS in the ejaculate. Many chemical toxins have a negative impact on sperm function. For example, lead and cadmium are heavy metals that reduce antioxidative activity of seminal plasma and sperm motility (Tvrda *et al*., 2012).

## Impacts of oxidative stress on sperm

Harmful effects of OS on sperm function can occur in many ways, as excess ROS damages a variety of important molecules, including lipids (Gutteridge and Halliwell, 1990), proteins (Stadtman and Levine, 2000) and DNA (Richter *et al*., 1988).

## Lipid peroxidation

Sperm have high levels of polyunsaturated fatty acids in their plasma membrane, which are vulnerable to oxidative attack. Lipid peroxidation generates a variety of lipid metabolites, including lipid peroxyl radicals, alkoxyl radicals, malonaldehyde, 4- hydroxynonenal, and acrolein (Jones *et al*., 1979). Lipid peroxyl radicals destabilize the plasma membrane by abstracting hydrogen atoms from adjacent polyunsaturated fatty acids. This process generates lipid radicals that react with oxygen to produce lipid hydro- peroxide and hydroxides, which in turn propagate a lipid peroxidation chain reaction. Lipid hydroperoxide is cleaved out of the membrane by phospholipase 2 and is further reduced by glutathione-peroxidase to truncated phospholipids. These molecules stop the chain reaction but concurrently destabilize the membrane (Storey, 1997). This affects functionality of integral membrane proteins as ion channels, with consequences for membrane integrity and fluidity (Aitken *et al*., 1989; Niki *et al*., 2005). Both membrane fluidity and integrity are important for a sperm to fuse with an oocyte. High concentrations of lipid peroxides in sperm were negatively correlated with their ability to fuse with the oocyte (Aitken *et al*., 1989). In addition, lipid peroxidation is also known to reduce sperm motility. Mechanisms involve modulation of ion channel function by membrane structure alterations (Nishikawa *et al*., 1989; Lundbaek and Andersen, 1994) and adduct formation of lipid metabolites with flagellar axonemal proteins (Baker *et al*. 2015; Moazamian *et al*., 2015) and mitochondrial electron transport proteins. Modulation of mitochondrial proteins disrupts mitochondrial electron transport, resulting in an efflux of electrons. These in turn combine with oxygen in a vicious cycle to form additional ROS (Aitken *et al*., 2012).

## Protein modifications

Reactive oxygen species are also postulated to directly modify proteins by oxidizing amino acid residue side chains, cleavage of peptide bonds, and formation of covalent protein-protein cross links (Garrison *et al*., 1962; Schuessler and Schilling, 1984; Davies, 1987). Conformation or activity of proteins can be influenced by oxidation of, for example, thiol groups. The amino acid cysteine contains a thiol group, and consequently, many proteins can be affected. In sperm, the enzyme tyrosine phosphatase, which has an important role in sperm capacitation, has a thiol group and is hence vulnerable to oxidation by ROS. Tyrosine phosphatase activity is inhibited by oxidation of the sulfhydryl group, initiating a cascade and finally inducing capacitation and the acrosome reaction in the sperm cell. Prolonged oxidative stress can also lead to over-oxidation of thiol groups of protamines and thus induce hyper-condensation of DNA, which adversely affects function (De Lamirande *et al*., 1997).

## DNA damage

Free radicals damage DNA in numerous ways. Hydroxyl radicals bind to the double-bonds of DNA bases and abstract hydrogen from the deoxyribose sugar (Breen and Murphy, 1995). The abstraction of hydrogen from deoxyribose carbon causes strand breaks and base releases. The ROS attack on bases leads to numerous base alterations (Henle and Linn, 1997). One of the most abundant modifications is oxidation of guanine. Hydroxyl radical addition to aqueous solutions induces formation of 8-oxo-7,8-dihydro-20-deoxyguanosine and 2,6-diamino-5-formamido-4-hydroxypyrimidine in cells (Burrows and Muller, 1998; Cadet *et al*., 2003). The former is one of the most mutagenic lesions, since it causes transversion mutations due to its ability to pair with adenine as well as cytosine bases (Wood *et al*., 1990).

Protamines cover most of the DNA, but depending on the species, up to 50% of histones are retained. In bulls, 13% of the paternal genome is still bound to histones (Samans *et al*., 2014). The exact biological function of this retention is unknown, but it is hypothesised that retained histones are marking sets of genes preferentially activated during early embryo development (Gardiner-Garden *et al*., 1998; Hammoud *et al*., 2009; Samans *et al*., 2014). These regions may be prone to DNA damage since they are less condensed (Noblanc *et al*., 2013).

## Repair mechanisms of DNA damage

Cells have diverse means of repairing DNA damage, including nucleotide excision repair, base excision repair, mismatch repair, and DNA double strand break repair (Evans and Cooke, 2009). In mature sperm, capacities for repairing DNA damage are limited. They only express one enzyme of the base excision repair pathway, 8-oxoguanine DNA glycosylase. Downstream components of the base excision repair pathway, apurinic endonuclease 1, and XRCC-1 (X-ray repair complementing defective repair in Chinese hamster cells 1) were not detectable (Smith *et al*., 2013). Consequently, mature sperm were reported to lack the ability to repair paternal DNA damage.

## Impacts of sperm damaged by oxidative stress on embryonic development

**Figure 2:**
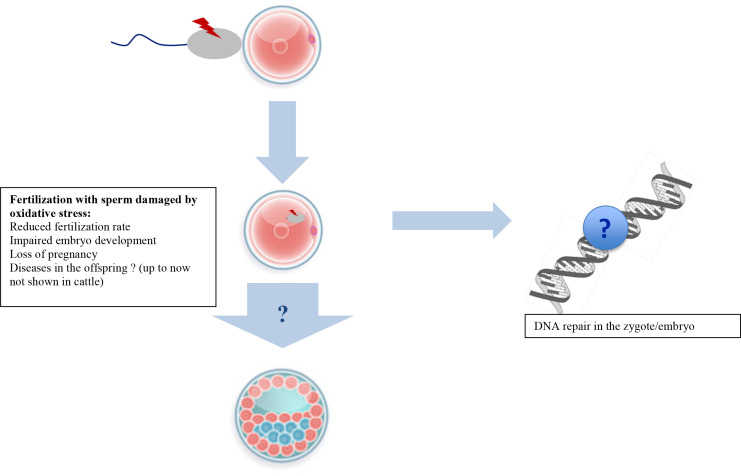
Effects of fertilization with sperm damaged by oxidative stress on embryo development.

There is no doubt that damage in the sperm can impair fertility and disrupt embryo development (Fig. 2). High levels of oxidative stress induce sperm plasma membrane alterations and hamper motility with the consequence of failure of fertilization. However, at lower levels of ROS, sperm may retain their ability to fertilize oocytes (Aitken and Baker, 2006). Through fertilization, not only the haploid paternal genome is transferred into the oocyte, but the entire content of the sperm, which may contain damaged molecules or toxic metabolites. Much of the content is disassembled, as it is not required for development (Cummins, 2001; Krawetz, 2005). Sperm mitochondria, for example, are degraded by the ubiquitination system (St John *et al*., 2000). However, other components of paternal origin are stable and have been followed until much later in embryonic development, including DNA, centrioles, some transcription factors, signalling molecules, and even ribonucleic acid (RNA) (Shalgi *et al*., 1994). Consequences of oxidative damage of paternal-derived molecules are extensively described for DNA. Effects of oxidative modifications in paternally derived centrioles or cytoplasmic factors on embryonic development have not been investigated. However, errors in microtubule assembly resulted in human fertilization failure and may contribute to a form of male infertility (Asch *et al*., 1995; Cummins, 2001).

Several studies investigated developmental consequences of sperm DNA damage induced by oxidative stress. Aitken and Baker (2006) demonstrated that exposure of sperm to low levels of hydrogen peroxide only marginally affected oocyte-sperm fusion, despite causing substantial DNA damage in sperm (Aitken and Baker, 2006). Thus using oxidatively damaged sperm for embryo production can lead to numerous developmental abnormalities. Low levels of pro-oxidants in bovine sperm cells had negative effects on blastocyst formation, but not on cleavage. Exposure of sperm to more severe oxidative stress reduced the blastocyst rate, cleavage rate and embryo quality (Silva, 2007; de Assis *et al*., 2015; De Castro *et al*., 2016). Simões *et al*. (2013) classified semen samples according to their sensitivity to OS and reported that increased susceptibility of sperm to OS compromised sperm DNA integrity and consequently reduced embryo quality.

In addition to negative effects on pre- implantation development, it is widely accepted that damaged sperm can support embryo development, implantation, and even pregnancy up to term, although development may be severely impaired. To the best of our knowledge, there are no reports in cattle on this topic. In mice, following IVF of mice using hydrogen peroxide damaged sperm, embryos developed, but were less likely to implant, were lighter, had a smaller crown- rump length, and female fetuses had metabolic abnormalities (Lane *et al*., 2014). In human assisted reproduction, fertilization with oxidatively damaged sperm, especially in regard to DNA damage, has been associated with loss of pregnancy or diseases in the offspring (Gavriliouk and Aitken, 2015).

As sperm cannot repair their genome before fertilization, DNA repair in newly fertilized embryos relies entirely on messenger ribonucleic acid (mRNA) and proteins stored in the oocyte (Ashwood-Smith and Edwards, 1996). It is suggested that a newly fertilized oocyte can cope with at least 10% of sperm DNA damage. This was derived from a study on trout, in which sperm with more than 10% tail DNA (based on a COMET assay) produced embryos, suggesting damage was either repaired or tolerated (Pérez-Cerezales *et al*., 2010).

Over 150 DNA repair genes have been identified in humans (reviewed by Jaroudi and SenGupta, 2007). Most belong to one of the four main DNA damage signaling and repair pathways: nucleotide excision repair, base excision repair, mismatch repair, and DNA double strand break repair. One of the earliest steps for DNA double-strand break repair is phosphorylation of the histone H2AX, referred to as gammaH2AX, which recruits DNA repair proteins (Celeste *et al*., 2003). Many DNA repair pathways are already active in early developmental stages. DNA damage repair pathways during early development interact with cell cycle progression. A cell with a damaged genome has three choices; remove the lesion, survive despite the lesion (with potential functional consequences), or undergo cell death. The importance of these repair mechanisms has been demonstrated by Barton (2007), who has used damaged sperm for fertilization or blocked DNA repair pathways in the zygote. When male rats were treated with cyclophosphamide, known to induce DNA damage, zygotes had enhanced gammaH2AX staining in the male pronucleus, compared to the control. In addition, Poly (ADP-Ribose) polymerase 1 (an enzyme which mediates DNA single-strand break repair in the base excision repair pathway) was upregulated in both pronuclei after fertilization with damaged sperm (Barton, 2007).

Rahman *et al*. (2012) studied the influence of oocyte quality on embryo production with damaged sperm in cattle and reported that bovine oocytes with a larger diameter were able to support embryo development after fertilization of sperm incubated in media with hydrogen peroxide better than their smaller counterparts. However, factors other than oocyte size can also influence oocytes repair capacity.

## Conclusions

Bovine sperm are exposed to OS under different conditions, causing damage to various cell structures. In particular, damage to sperm DNA can affect embryo development and increase embryo mortality. Whether disturbances of embryo development affect postnatal health of cattle, documented for other species, has apparently not been reported. It is well established that exogenous antioxidants can reduce negative effects of oxidative stress on sperm function and embryo development. However, there is still a knowledge gap regarding how oxidative stress can be avoided without inhibiting essential physiological effects of reactive species on fertilization.
